# Pancreatic Islet Xenograft Survival in Mice Is Extended by a Combination of Alpha-1-Antitrypsin and Single-Dose Anti-CD4/CD8 Therapy

**DOI:** 10.1371/journal.pone.0063625

**Published:** 2013-05-22

**Authors:** Efrat Ashkenazi, Boris M. Baranovski, Galit Shahaf, Eli C Lewis

**Affiliations:** Ben-Gurion University of the Negev, Faculty of Health Sciences, Department of Clinical Biochemistry and Pharmacology, Be’er Sheva, Israel; Virginia Commonwealth University, United States of America

## Abstract

Clinical pancreatic islet transplantation is under evaluation for the treatment of autoimmune diabetes, yet several limitations preclude widespread use. For example, there is a critical shortage of human pancreas donors. Xenotransplantation may solve this problem, yet it evokes a rigorous immune response which can lead to graft rejection. Alpha-1-antitrypsin (AAT), a clinically available and safe circulating anti-inflammatory and tissue protective glycoprotein, facilitates islet alloimmune-tolerance and protects from inflammation in several models. Here, we examine whether human AAT (hAAT), alone or in combination with clinically relevant approaches, achieves long-term islet xenograft survival. Rat-to-mouse islet transplantation was examined in the following groups: untreated (n = 6), hAAT (n = 6, 60–240 mg/kg every 3 days from day −10), low-dose co-stimulation blockade (anti-CD154/LFA-1) and single-dose anti-CD4/CD8 (n = 5–7), either as mono- or combination therapies. Islet grafting was accompanied by blood glucose follow-up. In addition, skin xenografting was performed in order to depict responses that occur in draining lymph nodes. According to our results hAAT monotherapy and hAAT/anti-CD154/LFA-1 combined therapy, did not delay rejection day (11–24 days untreated vs. 10–22 day treated). However, host and donor intragraft inflammatory gene expression was diminished by hAAT therapy in both setups. Single dose T-cell depletion using anti-CD4/CD8 depleting antibodies, which provided 14–15 days of reduced circulating T-cells, significantly delayed rejection day (28–52 days) but did not achieve graft acceptance. In contrast, in combination with hAAT, the group displayed significantly extended rejection days and a high rate of graft acceptance (59, 61, >90, >90, >90). In examination of graft explants, marginal mononuclear-cell infiltration containing regulatory T-cells predominated surviving xenografts. We suggest that temporal T-cell depletion, as in the clinically practiced anti-thymocyte-globulin therapy, combined with hAAT, may promote islet xenograft acceptance. Further studies are required to elucidate the mechanism behind the observed synergy, as well as the applicability of the approach for pig-to-human islet xenotransplantation.

## Introduction

Islet transplantation can provide type 1 autoimmune diabetes patients with functional islets and physiological circulating glucose levels (reviewed in [1]). However, shortage of human donors represents a critical obstacle [2]. Islet xenograft transplantation from non-human donors provides an alternative for human islet allotransplantation; in addition to providing abundant islet sources, xenografts offer a window of opportunity for genetically engineering donor cells towards superior islet function. However, the xenoimmune response is exceptionally rigorous, and the immunosuppression required might outweigh its benefits [2], [3].

Xenograft rejection is largely attributed to vast antigen disparity between species [4]. In addition, the process displays unique arms of the immune system to those that predominate in alloimmunity. For example, host CD4^+^ T-cells mediate the predominant injurious reaction to the islets, as mediated by local macrophages; in addition, evidence suggests that CD8^+^ T-cells [5] and B cells [6] partake in xenograft rejection. With some similarity to allograft rejection, local inflammation also limits islet xenograft survival, particularly in early days post-transplantation [7], [8], [9], a challenging obstacle considering that anti-inflammatory corticosteroids are diabetogenic and are excluded from current islet transplantation protocols.

Experimentally, xenograft survival prolongation has been achieved by several routes. Approaches that deplete immune cells have been mostly successful. Anti-thymocyte-globulin (ATG), a regimen comprised of polyclonal antibodies that temporarily deplete T-cells [10], is currently used for prevention of acute rejection in organ transplantation [11]. Combination of anti-CD4 and anti-CD8 antibodies in mice (referred to herein as T-cell debulking therapy) may represent the equivalent of ATG [12], [13]. Temporal T-cell depletion delays clonal T-cell activation in the associated draining lymph nodes (DLN) and allows grafted islets to evade T-cell-mediated destruction in the first two weeks post-transplantation. Indeed, anti-CD8 and anti-CD4 antibodies extend islet xenograft survival in experimental models [5].

In addition to T-cell depletion, co-stimulation blockade represents a successful approach for prolongation of xenograft survival. Since co-stimulation is required for T-cell activation [14], blockade of co-stimulatory signals has been widely employed. For example, monotherapy with anti-CD154 and anti-LFA-1 antibodies, as separate entities or together, prolonged xenograft survival [15], [16]. Muller Y et al. showed that combined anti-CD154 antibody and rapamycin induced Treg-mediated graft protection in rat-to-mouse islet xenotransplantation [17]. Inflammation blockade exerts favorable outcomes in islet transplantation [18], [19], [20], [21], [22], [23]. For example, human α1-antitrypsin (hAAT), a readily available plasma-derived glycoprotein with anti-inflammatory and tissue-protective attributes, promotes islet allograft survival and induces strain-specific immune tolerance in wild-type strains as well as in the non-obese diabetic (NOD) mouse model [18], [19], [23], [24]. hAAT also targets anti-islet autoimmune responses in animals [25]. The cellular targets of hAAT include non-T-cells such as dendritic cells [19], B lymphocytes [26], [27], macrophages and neutrophils [28], resulting in reduced levels and activity of inflammatory mediators such as IL-1β, tumor necrosis factor (TNF) α, monocyte chemotactic protein (MCP)-1 and nitric oxide, as well as elevating levels of IL-10 and IL-1 receptor antagonist [23], [29]. Specifically, hAAT has been shown to protect islets from inflammatory injury [23], [30], apoptosis [31] and isolation-related damage [32].

Based on the outcomes of hAAT therapy in allogeneic islet transplant models, we sought to examine whether hAAT therapy can be extended to modify the immune response that follows xenotransplantation in favor of islet xenograft acceptance and possible immune tolerance. We employed hAAT-transgenic mice that express hAAT in lung epithelia, and can accept multiple clinical-grade hAAT injections [18], [19], [33], [34], [35]. In addition, the possibility that hAAT may benefit synergistically from the addition of a supportive immunosuppressive approach was explored.

## Methods

### Animals

Six-to-eight-week old C57BL/6 mice (Harlan laboratories Inc., Israel) and hAAT lung-specific transgenic mice (C57BL/6 background, kind gift from Prof. A. Churg, University of British Columbia, Vancouver, Canada [33]) were used as graft recipients. Nine-to-ten-week old Sprague Dawley female rats (Harlan laboratories) were used as pancreatic islet and skin donors. Experiments were approved by the Ben-Gurion University of the Negev Animal Care and Use Committee (Permit Number IL-12-01-2009). All efforts were made to minimize suffering of the animals.

### Pancreatic Islet Isolation

Donor rats were anesthetized and then bled. The bile duct was ligated at the liver and at the intestinal ends, then cannulated with a 27G needle. The pancreas was inflated with 10 ml cold collagenase (1 mg/ml, type XI, Sigma, Israel), removed and incubated for 17 minutes at 37°C while continuously stirred with a 3 mm sterile magnet. Digested pancreas was mechanically sheared by vortex and tissue was filtered through a 1,000 µm sieve. Islets were collected from a double-Ficoll gradient (1.0771 and 1.1191, Sigma). The resulting material was washed in Hanks balanced salt solution (HBSS) containing 0.5% bovine serum albumin (BSA) (cell-culture tested, Sigma), centrifuged at 900 revolutions per minute (rpm) and then reconstituted in culture medium containing RPMI-1640, 10% fetal calf serum (FCS) (both from Biological Industries, Beit Haemek, Israel), 50 units/ml penicillin and 50 µg/ml streptomycin (both from Cellgro, Mediatech, Herndon, VA, USA). Pancreatic islets were then hand-picked under a stereoscope into a culture flask and incubated overnight.

### Islet Xenotransplantation

Islet transplantation in the renal subcapsular space was performed as described, with minor modifications [19]. Rat islets (400/transplant) were implanted under the renal capsule of recipient mice that were rendered hyperglycemic by single-dose streptozotocin (225 mg/kg, Sigma). Prospective recipients were screened for non-fasting circulating glucose levels of ∼400 mg/dl. Blood glucose was followed three times a week, and graft failure was determined by glucose values exceeding 300 mg/dl after at least three days of normoglycemia.

### Skin Xenotransplantation

Skin transplantation was performed as described [19] with minor modifications. Donor rats were anesthetized, abdominal midline was shaved and excised skin was placed in cold phosphate-buffered saline (PBS). Blood vessels and hypodermis were removed using sterile blade and the skin was cut into 1 mm^2^ pieces under a stereoscope. Grafts were implanted subcutaneously in the inner-thigh region of recipients and incision sites were stitched closed.

### Treatment Protocols

hAAT (Glassia™, Kamada, Israel) was introduced at 60 and 240 mg/kg, intraperitoneally (i.p.) and at either 1 or 10 days prior to transplantation. Therapy continued every 3 days throughout the experiments, as described [19]. The maximal treatment duration was 80 days. Temporary T-cell depletion (debulking therapy) included a single dose of a mixture of depleting polyclonal anti-CD4 (GK1.5) and anti-CD8 (53.6.72) antibodies (BioXCell), each at 300 µl at the concentration of 1 mg/ml, 3 days prior to transplantation. Subtherapeutic co-stimulation blockade included an equal mixture of anti-LFA-1 and anti-CD154 monoclonal antibodies (MR-1 and FD441.8, respectively, BioXCell, West Lebanon, NH, USA), each at 25µl/injection at the concentration of 1.25 mg/ml, one day before transplantation and every three days thereafter. The maximal treatment duration was 40 days.

### Histology and Immunohistochemistry

Explanted kidneys carrying implants were fixed in 10% formalin (Sigma) for 24 h and transferred into 70% ethanol. The specimens were cut through the center of the implant, embedded in paraffin and sectioned. For histological examination, Hematoxylin and Eosin (H&E) was performed. Insulin immunostaining was performed with guinea-pig-anti-swine-insulin, detected by Cy3-donkey-anti-guinea-pig (both 1∶200, DakoCytomation, Glostrup, DK); B cell immunostaining was performed with rat-anti-mouse-B220 (1∶100, eBioscience, San-Diego, CA, USA), detected by DyLight488-goat-anti-rat (1∶200, Jackson IR, PA, USA); T-cell immunostaining was performed with Armenian-hamster-anti-CD3 (BioLegend, San-Diego, CA, USA), detected by fluorescence isothiocyanate (FITC)-rat-anti-Armenian-hamster (eBioscience), both at 1∶50; Treg immunostaining was performed with mouse-anti-mouse-foxp3 (Biolegend), detected by Cy2-donkey-anti-mouse (Jackson IR), both at 1∶100. Nuclei were depicted by 4′,6-diamidino-2-phenylindole (DAPI) staining (1 µg/ml, Sigma). Immunofluorescence was detected using Olympus BX60 (Olympus UK Ltd., London, UK).

### Reverse Transcriptase-polymerase Chain Reaction (RT-PCR)

Total RNA was extracted from DLN or implants using RNA extraction kit (5 Prime Perfect Pure RNA Tissue Kit, MD, USA). Reverse transcription was performed using Verso complementary DNA (cDNA) Kit (Thermo scientific UK). cDNA amplification was undertaken by PCR (XP Cycler, BIOER) set at 28–43 cycles, depending on gene expression intensity. The results were collected from a series of at least 3 different cycles, normalized to β-actin and calculated as fold from control. Species-specific primers included (forward 5′ to 3′; reverse 5′ to 3′): ***Mouse*** *:* **β-actin** GGGTCAGAAGGATTCCTATG; GGTCTCAAACATGATCTGGG, **CD3** GCCTCAGAAGCATGATAAGC; CCCAGAGTGATACAGATGTC, **CD14** GCCTCAGAAGCATGATAAGC; CCCAGAGTGATACAGATGTC, **IL-1β** CTCCATGAGCTTTGTACAAGG; TGCTGATGTACCAGTTGGGG, **CD86** TCCAGAACTTACGGAAGCACCCACG; CAGGTTCACTGAAGTTGGCGATCAC, **CD40** ATTTGTGCCAGCCAGGAAGCCG; GCATCCGGGACTTTAAACCACAGA, **IL-6** CTGGGAAATCGTGGAAATGAG; GTTAGGAGAGCATTGGAAATTGG, **IL-10** AGGACTTTAAGGGTTACTTGG; CTATGCAGTTGATGAAGATGTC, **B220** CCTTTGTGATGAGTTACTGGA; CCTTCCTCTTGGAATGTCTC, **LY94** GTCACAAATGGAAACTCGGT; TCATACAGACCAGTTACTACCAG. ***Rat*** *:* **β-actin** GGCTTTAGGAGCTTGACAATACTG; GCATTGGTCACCTTTAGATGGA, **insulin** GCAAGCAGGTCATTGTTCC; TGCCAAGGTCTGAAGATCC, **MCP-1** CTGCTGCTACTCATTCACTG; CTTGGTGACAAATACTACAGCT.

### In vitro Islet Stimulation

Rat pancreatic islets (50/well in 48-well plates in triplicate) were cultured with medium alone or with recombinant IL-1β (10 ng/ml, R&D Systems), in the presence or absence of a 1 h pretreatment with hAAT (0.5 mg/ml). Nitrite concentration was determined after 72 h by Griess assay (Promega, WI, USA).

### FACS Analysis

Percent CD3^+^ cells out of circulating CD45^+^ leukocytes was determined in fresh heparinized whole blood obtained from mouse-tails. Red blood cells (RBC) were lysed using RBC lysis buffer followed by double-staining with FITC-anti-CD3 (BD Biosciences) and APC-anti-CD45 (eBioscience). Each sample contained at least 1×10^6^ cells. Percent B cells in DNL were determined in single-cell suspensions of excised lymph nodes. Triple-staining was preformed using phycoerythrin (PE)-anti-CD40, FITC-anti-CD19 and APC-anti-B220 antibodies (all from eBioscience and diluted according to manufacture’s recommendation). FACS analysis was carried out using FACS Calibuer (Becton Dickinson). Data was analyzed using Cell Quest software.

### Statistical Analysis

GraphPad Prism 5 (Pugh computers, UK) was used for computerized statistical analysis. Results are expressed as the mean ± standard error of the mean (SEM). Significance of differences between groups was determined by two-tailed student *t*-test at 95% confidence interval. Survival was analyzed by Kaplan–Maier analysis. Means were considered statistically different at *p*<0.05.

## Results

### hAAT Monotherapy during Rat-to-mouse Islet Transplantation

**Figure 1:**
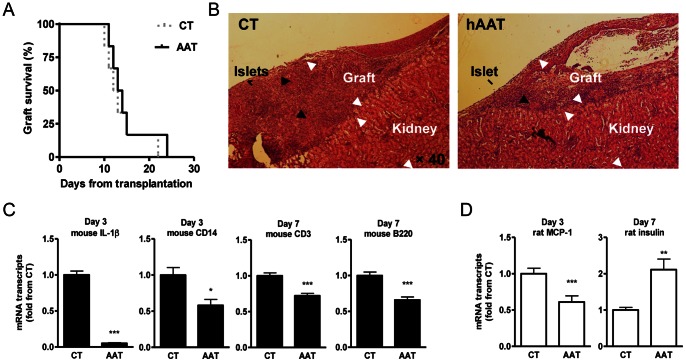
Human AAT monotherapy during pancreatic islet xenotransplantation. Rat pancreatic islets were grafted into the renal subcapsular space of hyperglycemic mice. Recipients were treated with saline (CT) or human AAT throughout the experiment. (**A**) Islet graft survival curve (*n* = 6/group). (**B**) Graft histology. Representative day-seven explanted grafts from CT and hATT-monotreated mice (*n* = 3/group). *Black arrows*, remains of rat pancreatic islets. (**C**) Mouse gene expression at graft site. Grafts were explanted at indicated times after transplantation. Mean ± SEM from *n* = 3 grafts/group; *p<0.05, **p<0.01, ***p<0.001. (**D**) Rat gene expression at graft site. Grafts were explanted at indicated times after transplantation. Mean ± SEM from *n* = 3 grafts/group; **p<0.01.

In all experiments, the hAAT group is comprised of recipient mice that are transgenic for lung-derived hAAT. The initial dose for hAAT monotherapy (60 mg/kg from 1 day prior to transplantation) was selected from previous reports [36]. In order to explore a monotherapy protocol with a higher impact, both a higher dose (240 mg/kg) and an extended 10-day pretreatment protocol were tested. These doses are within the clinically used range in patients [36]. hAAT injections were repeated every 3 days in all experiments in accordance with the turnover of human AAT in mice [23]. A total of *n* = 6 mice were grafted under these conditions, including two recipients per modified protocol. In addition, *n* = 6 mice were grafted with no added therapy, as control. As shown in Figure 1A, neither of the three dose-modified hAAT monotherapy protocols (the pooled outcomes of initial dose, high-dose and extended high-dose hAAT protocols) delayed islet xenograft rejection day (untreated mice rejected on days 10,11,12,13,15, 22 and pooled hAAT-treated mice rejected on days 11, 12, 13, 14, 15, 24). The extended hAAT protocol is used throughout the following studies.

Intragraft changes were examined (Figure 1 B–D). According to histology on day 7 post-transplantation (*n* = 3 per group, representative images), infiltrate and various degrees of islet remains appeared similar between groups (Figure 1B). Rat and mouse gene expression levels were examined on days 3 and 7 post-transplantation (*n* = 3 for each group and time-point). The expression of mouse IL-1β significantly decreased 20-fold on average in the hAAT-treated group. Mouse CD14 decreased by 1.72 on average, as did infiltrating CD3 and B220 transcripts. Rat MCP-1 decreased by 1.74 on average and insulin transcript levels increased 2.1-fold (Figure 1 C–D). No significant differences were observed in the expression of mouse LY94, a natural killer (NK) cell marker (not shown). According to insulin immunohistochemistry of grafts from 3 days post-transplantation (not shown), islets appeared partially damaged morphologically and nuclear staining revealed infiltration of cells around islets in both groups.

Since a concern was raised regarding potential differences between mouse and rat cell response to hAAT, rat islets were incubated with stimulatory concentrations of IL-1β and the release of nitric oxide determined; as expected, in the presence of hAAT (0.5 mg/ml) there was a decrease in IL-1β-stimulated nitric oxide release by 30% (not shown), indicating that rat islets respond to hAAT in a comparable manner to mouse islets [23].

### DLN Molecular Profile during hAAT Monotherapy

**Figure 2:**
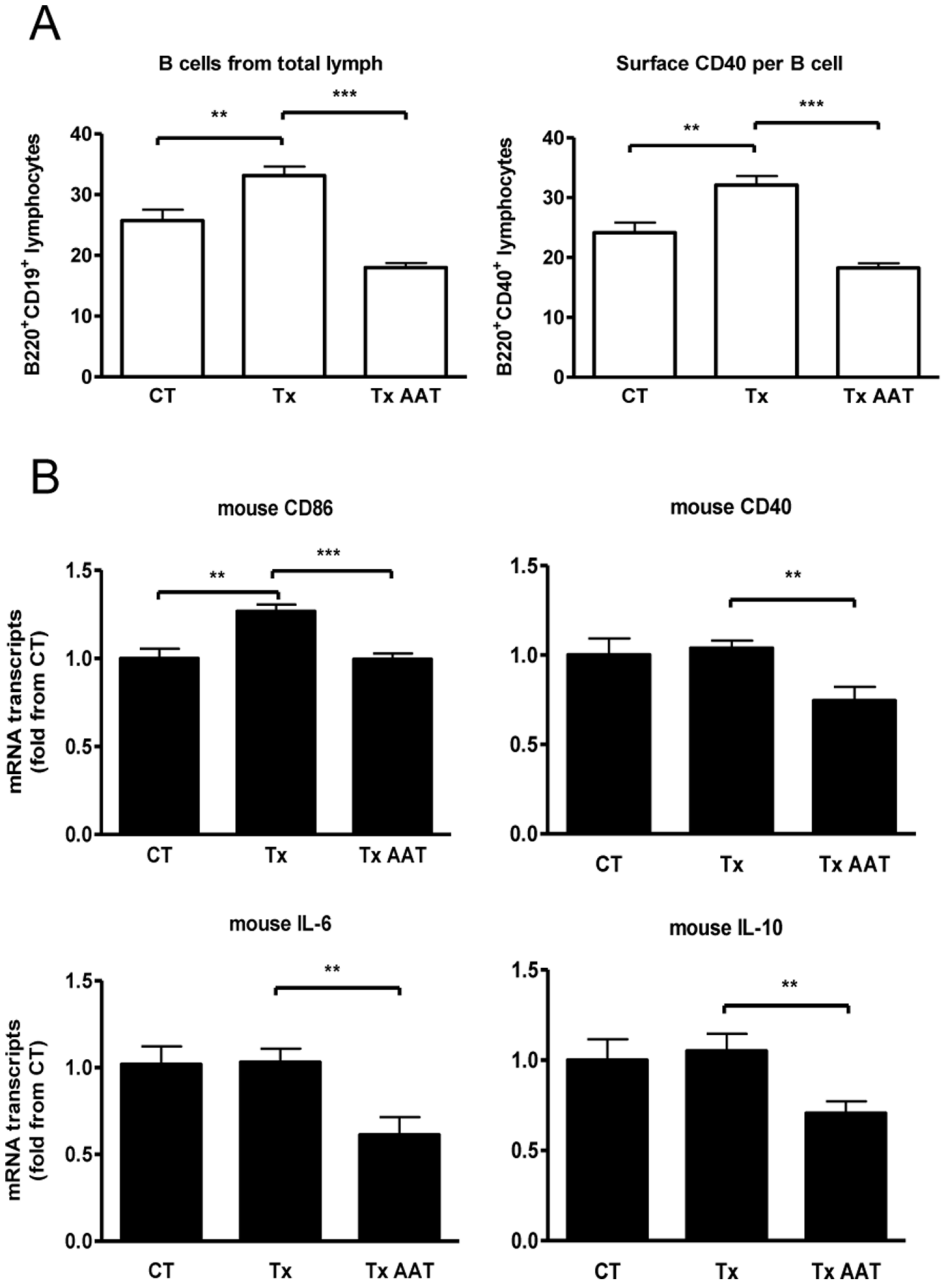
DLN response to human AAT monotherapy after skin xenografting. Mice were either SHAM operated (CT) or recipients of rat skin (Tx) in the absence or presence of human AAT monotherapy. (**A**) 14-day DLN. FACS analysis. Results expressed as fold change from CT, mean ± SEM from n = 10/group; **p<0.01, ***p<0.001. (**B**) 72-h DLN. RT-PCR. Results expressed as fold change from CT, mean ± SEM from *n* = 3/group; **p<0.01, ***p<0.001.

In order to achieve a robust immune response and improve detection of changes in DLN, as well as to encourage responses with low variability, skin xenotransplantation was performed. Treatment groups included control mice and mice receiving hAAT. Day-14 inguinal DLN were collected for FACS analysis. As shown in Figure 2A, the number of B cells in the lymph nodes rose by 22.4% on average in transplanted mice, compared to control non-grafted mice. However, hAAT-treated mice displayed a 54.2% decrease on average of B cells from skin transplanted untreated mice. In addition, surface levels of CD40 significantly increased compared to non-grafted mice, and then reduced with hAAT treatment.

DLN RT-PCR analysis was performed 3 days after transplantation. Figure 2B depicts relative changes in specific transcript numbers. While DLN CD40, IL-6 and IL-10 transcript levels did not increase after xenotransplantation at this time point, CD86 displayed a significant increase from non-grafted mice. In the presence of systemic hAAT, CD40 was reduced by 28.3% on average, CD86 by 21.5%, IL-6 by 40.6% and IL-10 by 32.87%.

### Islet Xenotransplant Survival is Extended under hAAT and Temporary T-cell Depletion Combination

Since monotherapy with hAAT appears to have allowed an uninterrupted xeno-response, we sought to examine whether the combination of hAAT treatment with another approach for modifying immune responses, namely, temporary T-cell depletion, might exhibit a synergistic behavior.

**Figure 3:**
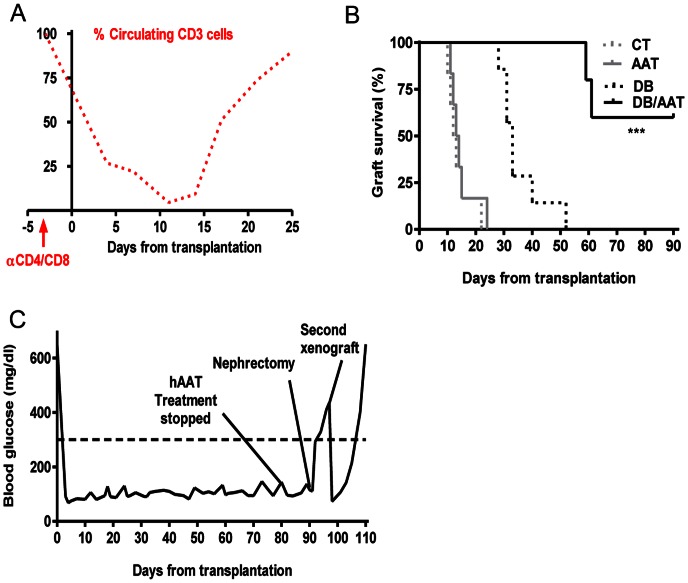
AAT treatment combined with debulking therapy; graft survival. **(A–C)** Rat islets were grafted into mice that were treated with anti-CD4/CD8 depleting antibodies, in the absence of AAT therapy (*n* = 7) or with added AAT therapy (*n* = 5). (**A**) CD45^+^CD3^+^ cells from peripheral blood, as monitored by FACS analysis. Results presented as the percent out of initial amount prior to injection. Representative follow-up out of 10 mice. (**B**) Islet xenograft survival curve. ***p<0.001 between DB and BD/AAT. (**C**) Glucose follow-up. Representative mouse. Milestones indicated: *hAAT treatment stopped,* therapy withdrawn followed by glucose follow-up; *nephrectomy*, graft explantation followed by glucose follow-up; *second xenograft*, rat islets grafted into the right renal subcapsular space followed by glucose follow-up.

**Figure 4:**
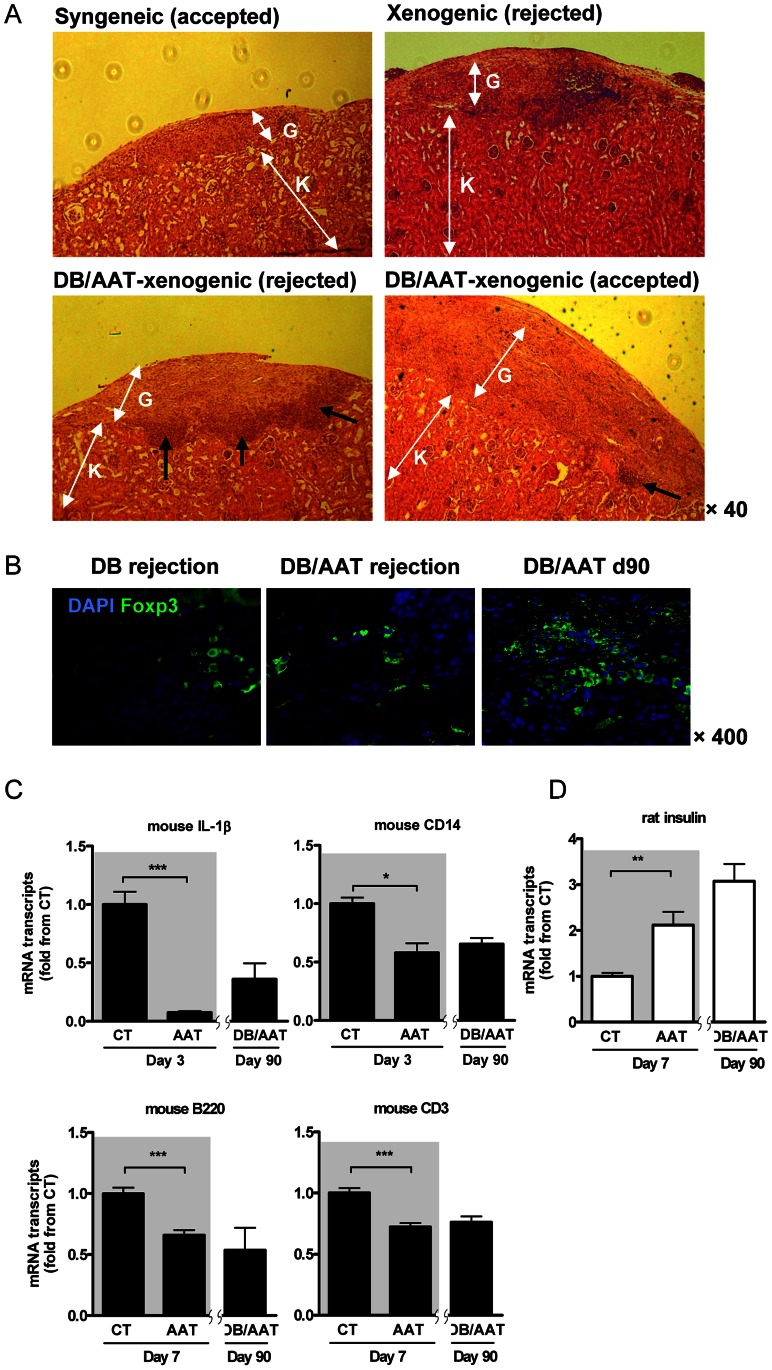
AAT treatment combined with debulking therapy; histology and gene expression. Rat islets were grafted into mice that were treated with anti-CD4/CD8 depleting antibodies, in the absence of AAT therapy (*n* = 7) or with added AAT therapy (*n* = 5), as in Figure 3B. (**A**) Graft site histology. *K*, kidney tissue; *G*, graft site. From *left* to *right*, representative syngeneic mouse islet graft (day 35), xenograft (debulking therapy alone, day 25), *black arrows* indicate immune cell mononuclear infiltration, xenograft (debulking therapy combined with AAT, day 11 after rejection) and xenograft (debulking therapy combined with AAT, day 90). (**B**) Treg cell content in xenograft sites. Immunofluorescent staining. DB, debulking therapy alone (rejected graft); DB/AAT, combined debulking and AAT therapy (rejected and accepted grafts). *Green*, foxp3; *blue*, DAPI nuclear counterstaining. Representative images. (**C**) Mouse (recipient) gene expression profiles. RT-PCR. CT vs. AAT monotherapy, see Figure 1C, shown over gray background, next to day 90 explants from mice treated by the combination of debulking therapy and AAT (DB/AAT). Results expressed as fold change from CT, mean ± SEM from *n* = 3/group; *p<0.05, **p<0.01, ***p<0.001. (**D**) Rat (donor) insulin expression profile. RT-PCR. CT vs. AAT monotherapy, see Figure 1D, are shown over gray background, next to day 90 explants from mice treated by the combination of debulking therapy and AAT (DB/AAT). Results expressed as fold change from CT, mean ± SEM from *n* = 3/group; **p<0.01.

Debulking therapy was examined alone and in combination with hAAT (Figure 3 A–C and Figure 4). Recipient mice were treated with single-dose anti-CD8/CD4 depleting antibodies, with or without hAAT (*n* = 5–7 per group). According to circulating mouse CD45^+^CD3^+^ follow-up (Figure 3A, representative result), mice injected with depleting antibodies exhibited a decrease in the relative number of circulating T-cells and a spontaneous return to normal lymphocyte levels after a period of approximately two weeks.

As shown in Figure 3B, animals treated by debulking therapy (DB) displayed a delay in xenograft rejection (days 28, 31, 31, 33, 33, 40, 52). In contrast, combined debulking therapy with hAAT (DB/AAT) resulted in islet xenograft surviving until days 59, 61, >90, >90, >90. In addition, a group of animals was examined for the outcome of combined debulking therapy with a lower dose of hAAT (60 mg/kg, n = 6, not shown). Three out of 6 recipients displayed rejection days at the range of debulking therapy alone and three were extended beyond these time periods (22, 29, 32, 74, 83, >84).

In order to assess whether combined debulking therapy and hAAT promotes strain-specific immune tolerance, islet grafts were recovered from long-lasting recipients (*n* = 3), and mice were allowed to return to hyperglycemic values. A second graft of rat islets was placed under the right renal capsule. As shown in Figure 3C (representative glucose follow-up), acute rejection was observed.

### hAAT and Temporary T-cell Depletion Combination Results in Modified Graft Site Immune Infiltration and Gene Expression Profiles

As previously reported using an allogeneic islet transplant model [18], [19], hAAT monotherapy results in a non-invasive population of mononuclear cells that is located in the region between the renal tissue, capsule and graft, containing Tregs. Here, we compared histological images of islet grafts that lack an immune infiltrate (syngeneic mouse islet transplants) with histological samples collected from untreated xenogenic grafts, as well as xenogenic transplants treated by combination of debulking therapy and hAAT that were either accepted or rejected. As shown in Figure 4A (representative histological images), 35-day syngeneic islet graft sites are characterized by lack of an immune infiltrate and untreated xenotransplants displayed robust infiltration throughout the graft site (10 days after rejection).

Histology obtained from treated mice was divided into two: *bottom left*, a graft that was rejected on day 59 and examined 11 days later, and *bottom right*, a graft that was accepted (obtained 90 days post-transplantation). The rejected graft presented with a marginal mononuclear cell infiltrate that was not limited to the region between capsule, graft and kidney, but rather appeared to line the border with the host (black arrows). In contrast, an accepted xenograft displayed a restricted infiltrate adjacent to the capsule and consistent with that found in long-term allogeneic hAAT-treated islet transplants.

### hAAT and Temporary T-cell Depletion Combination Decreases T and B Lymphocyte Content in Xenografts and Promotes Local Foxp3^+^ Tregs

Explanted grafts were analyzed for T and B cell markers, as well as for Tregs by immunohistochemistry. As shown in Figure 4B, representative images from grafts: debulking therapy 10 days after rejection, DB/AAT 11 days after rejection and DB/AAT that did not reject. Foxp3-positive Tregs were abundant in the accepted grafts. In addition, populations of CD3^+^ and B220^+^ cells were reduced in both debulking alone and combined debulking and hAAT, compared to untreated animals (not shown).

### hAAT and Temporary T-cell Depletion Combination Affects Intragraft Gene Expression Profile

Since the majority of grafts treated solely by T-cell debulking did not survive beyond day 30, gene expression was examined between samples from day-7 untreated (CT) or hAAT-treated (AAT) xenografts (shaded gray) and day-90 combination therapy (DB/AAT) (Figure 4C). Days 3 and 7 results are also shown in Figure 1C, repeated here in order to facilitate visual appreciation of the comparison between the groups. As shown, combined treatment with hAAT and temporary T-cell depletion resulted in overall consistent transcript levels between both hAAT-treated groups on day-90. Rat insulin transcripts were greater in day-90 combined-therapy compared to both day-7 groups (Figure 4D).

### Islet Xenotransplants are Rejected under hAAT and Low-dose Co-stimulation Blockade Combination

Since combined treatment of hAAT and depleting antibodies resulted in extension of xenograft survival, hAAT with a combination of co-stimulation blockade was examined as another way for a possible xenograft survival. Mouse monoclonal anti-CD154 and anti-LFA-1 antibodies promote xenograft survival [15], [37]. Recipients were treated with low-dose co-stimulation blockade with or without hAAT (*n* = 6 per group). Treatment with sub-effective low-dose co-stimulation blockade alone displayed a rejection rate similar to that of control untreated recipient mice (days 10, 12, 12, 15, 15, 17). Similarly, combination of low-dose co-stimulation blockade and hAAT resulted in a non-significant change to outcomes of control or low-dose co-stimulation blockade alone; the grafts were rejected on days 10, 10, 13, 15, 15, 19, 37 (data not shown).

## Discussion

Attempts to allow islet xenografts to survive the rigorous xenoimmune response have shown some degree of success in various experimental systems. Here, we sought to evaluate clinically-relevant approaches alone and in combination, in order to find synergistic outcomes that may translate to clinical studies. The arms evaluated included inflammation blockade by hAAT, T-cell depletion by anti-CD4/CD8 depleting antibodies and co-stimulatory blockade by anti-CD154/LFA-1. The combination of temporary T-cell depletion with hAAT therapy extended islet xenograft survival and increased graft acceptance rate.

In the setup of xenografting, we exploited the possibility of RT-PCR for the detection of species-specific transcripts in the explants, a feat unattainable in allogeneic models due to overlap between graft and host transcript sequences. We were able to demonstrate that intragraft host-IL-1β expression was significantly reduced in the presence of hAAT, a change that favors islet survival [22], [38], [39], [40], [41], and that host-CD14 transcripts were reduced, reflecting, most likely, reduced host monocytic infiltration. Similarly, we found that graft-derived MCP-1 expression declined, a change shown to be beneficial in human islet transplantation [42]. Although descriptive in nature, the panel of changes in gene expression support the anti-inflammatory changes brought upon by hAAT.

Rat-to-mouse islet transplantation elicits a xenoimmune response that is not dissimilar to that of other xenograft models, representing to some extent the potential highly-sought pig-to-human xenotransplantation [17], [43], [44], [45]. Unlike higher mammals, this model allows one to perform multiple repeats, expand time schedules, employ multiple doses and explore genetically modified strains of mice. For example, the current study utilizes hAAT-transgenic mice in order to allow prolonged treatment with exogenous hAAT [19], [34].

According to literature, the number of rat pancreatic islets that are transplanted to a diabetic mouse and correct glucose levels greatly vary in literature, and most commonly range between 250 and 750 [46]. We found that transplantation of 400 islets achieves normoglycemia and the occurrence of graft rejection after 10–22 days.

Although monotherapy with hAAT protects islet allografts from acute rejection and facilitates strain-specific immune tolerance, hAAT monotherapy appears insufficient to allow islet xenograft acceptance. For example, histology on day 7 depicts a rather similar degree of infiltrate and islet remains, between the treated and untreated groups. The histology in this time point is variable in light of the proximity with the surgical procedure, thus we performed more quantitative measures to support the histology (Figure 1C and 1D). In an attempt to optimize the impact of hAAT within the experimental setup, we evaluated the effect of the highest clinically-relevant dose of hAAT [47], and also examined a 10-day pre-conditioning protocol, with the rationale that the anti-inflammatory profile of the recipient will be progressively enhanced. Nevertheless, these extensions of the original monotherapy protocol did not provide a significant change in graft acceptance rate, although intragraft gene expression profile appeared to share trends with previous findings that relate to allograft treatment, i.e., expression of inflammatory genes was diminished. Day-7 was chosen for gene analysis of adaptive immunity elements [6]. Thus, we found that hAAT decreased transcript levels of CD3 and B220 at the graft site, as supported by histology. Here, we utilized a skin xenograft model in order to evoke large measurable amplitudes of changes in the lymph nodes and show that hAAT treatment resulted in a smaller population of CD40-positive B cells in hAAT-treated mice, by FACS. The reduced levels of CD40 found here and in other reports [18], [19], [27] support the notion that hAAT might interfere with CD40-related responses, a pivotal pathway in B cell activation. Whether hAAT directly affects B cells is currently under investigation. In addition, the response to skin transplants is quite different than the corresponding response to islet transplants; thus, the data gathered here favor further investigation with the particular implementation of islet transplants.

We found no difference in local transcript levels of the NK cell marker, LY94. Little is known regarding the involvement of NK cells in islet transplantation [48], [49]; it is possible that day-3 is not ideal for examination of NK cells in the graft. Indeed, we have previously shown that NK infiltration towards allogeneic cell transplants is reduced by hAAT on days 4–5 [23]. More studies should be undertaken in order to examine the aspect of NK cells in hAAT-treated islet xenografts.

hAAT did not enhance the expression of IL-10. The lack of IL-10 also correlates with the absence of a strain-specific tolerance in the current study, and may represent a critical obstacle in xenograft acceptance. The background for this discrepancy is unclear.

In light of the failure of hAAT monotherapy to protect xenografts, despite mostly consistent trends in graft-site gene expression profile and dampened immune responses, it became apparent that there may be factors that govern the immune response to xenotransplantation that are outside the ability of hAAT to modulate. Thus, we extended the study to incorporate combination therapies.

Treatment with depleting anti-CD4/CD8 antibodies delayed rejection time, and combined with hAAT achieved a significant improvement in outcomes. Since AAT does not directly inhibit T-cell responses [19], [25], these finding suggest that hAAT may direct the immune response in the first stages post-transplantation in a manner that is compromised by the presence of uninterrupted activated T-cells. Therefore, we assume that the temporary elimination of T-cells together with hAAT, affords xenografts improved conditions for recovery and survival, and provides the re-emerging T-cells with diminished danger signals. It is also possible that the rare population of CD4^+^ or CD8^+^ dendritic cells were depleted [50]; both their response to hAAT and their involvement in xenograft rejection are currently under investigation.

Although combined treatment with depleting antibodies and hAAT did not lead to long-term immune tolerance, we show that there is a non-invasive cell infiltrate around the graft site, as obtained in allotransplantation in the presence of hAAT [19] and other approaches [51], suggesting that there is an element of graft tolerance. It would be interesting to investigate other safe combinations that promote Tregs, and examine whether they can provide the extra measure required to achieve tolerance in the current setup.

Since hAAT with combination of temporary T-cell depletion resulted in xenograft survival extension we tried another combined therapy known as co-stimulation blockade. The rationale for subtherapeutic doses of anti-CD154/LFA-1 antibodies relates to the finding in which this combination of antibodies alone can protect islet xenografts [14]. Indeed, the low-dose of co-stimulation blockade practiced here did not provide protection to grafts as a monotherapy. Interestingly, reports suggest that the effects of AAT involve reduced CD40 surface levels in professional antigen presenting cells [18], [19], rendering the combination, at best, additive.

In conclusion, the study demonstrates that combined temporal T-cell blockade and hAAT therapy, both clinically viable options, are able to significantly advance islet xenograft acceptance in the studied model. Since the mechanism of xenotransplantation is not fully understood, further research is needed to understand the entities that limit such success from reaching full impact. Further studies may both enhance our understanding of the mechanism behind the protective attributes of this clinically-safe approach, as well as to directly test its feasibility in pig-to-human islet xenotransplantation.

## References

[1] de Kort H, de Koning EJ, Rabelink TJ, Bruijn JA, Bajema IM (2011) Islet transplantation in type 1 diabetes. BMJ 342: d217. 21257658 10.1136/bmj.d217

[2] Sprangers B, Waer M, Billiau AD (2008) Xenotransplantation: where are we in 2008? Kidney Int 74: 14–21. 18418354 10.1038/ki.2008.135

[3] Jones PM, Courtney ML, Burns CJ, Persaud SJ (2008) Cell-based treatments for diabetes. Drug Discov Today 13: 888–893. 18652911 10.1016/j.drudis.2008.06.014

[4] Rayat GR, Johnson ZA, Beilke JN, Korbutt GS, Rajotte RV, et al (2003) The degree of phylogenetic disparity of islet grafts dictates the reliance on indirect CD4 T-cell antigen recognition for rejection. Diabetes 52: 1433–1440. 12765954 10.2337/diabetes.52.6.1433

[5] Koulmanda M, Laufer TM, Auchincloss H Jr, Smith RN (2004) Prolonged survival of fetal pig islet xenografts in mice lacking the capacity for an indirect response. Xenotransplantation 11: 525–530. 15479462 10.1111/j.1399-3089.2004.00174.x

[6] Kobayashi T, Harb G, Rajotte RV, Korbutt GS, Mallett AG, et al (2006) Immune mechanisms associated with the rejection of encapsulated neonatal porcine islet xenografts. Xenotransplantation 13: 547–559. 17059582 10.1111/j.1399-3089.2006.00349.x

[7] Korsgren O, Nilsson B (2009) Improving islet transplantation: a road map for a widespread application for the cure of persons with type I diabetes. Curr Opin Organ Transplant 14: 683–687. 19779341 10.1097/MOT.0b013e328332c44c

[8] van der Windt DJ, Bottino R, Casu A, Campanile N, Cooper DK (2007) Rapid loss of intraportally transplanted islets: an overview of pathophysiology and preventive strategies. Xenotransplantation 14: 288–297. 17669170 10.1111/j.1399-3089.2007.00419.x

[9] Barshes NR, Wyllie S, Goss JA (2005) Inflammation-mediated dysfunction and apoptosis in pancreatic islet transplantation: implications for intrahepatic grafts. J Leukoc Biol 77: 587–597. 15728243 10.1189/jlb.1104649

[10] Broady R, Yu J, Levings MK (2009) ATG-induced expression of FOXP3 in human CD4(+) T cells in vitro is associated with T-cell activation and not the induction of FOXP3(+) T regulatory cells. Blood 114: 5003–5006. 19822903 10.1182/blood-2009-04-214437

[11] Gaber AO, Monaco AP, Russell JA, Lebranchu Y, Mohty M (2010) Rabbit antithymocyte globulin (thymoglobulin): 25 years and new frontiers in solid organ transplantation and haematology. Drugs 70: 691–732. 20394456 10.2165/11315940-000000000-00000

[12] Tchorsh-Yutsis D, Zlotnikov Klionsky Y, Bachar-Lustig E, Aronovich A, Feine I, et al (2011) Embryonic pig pancreatic tissue for the treatment of diabetes: potential role of immune suppression with “off-the-shelf” third-party regulatory T cells. Transplantation 91: 398–405. 21192322 10.1097/TP.0b013e318204be15

[13] Tchorsh-Yutsis D, Hecht G, Aronovich A, Shezen E, Klionsky Y, et al (2009) Pig embryonic pancreatic tissue as a source for transplantation in diabetes: transient treatment with anti-LFA1, anti-CD48, and FTY720 enables long-term graft maintenance in mice with only mild ongoing immunosuppression. Diabetes 58: 1585–1594. 19401429 10.2337/db09-0112 PMC2699862

[14] Snanoudj R, Zuber J, Legendre C (2010) Co-stimulation blockade as a new strategy in kidney transplantation: benefits and limits. Drugs 70: 2121–2131. 20964456 10.2165/11538140-000000000-00000

[15] Arefanian H, Tredget EB, Rajotte RV, Korbutt GS, Gill RG, et al (2007) Combination of anti-CD4 with anti-LFA-1 and anti-CD154 monoclonal antibodies promotes long-term survival and function of neonatal porcine islet xenografts in spontaneously diabetic NOD mice. Cell Transplant 16: 787–798. 18087999 10.3727/000000007783465244

[16] Tredget EB, Arefanian H, Gill RG, Rajotte RV, Rayat GR (2008) Monotherapy with anti-LFA-1 monoclonal antibody promotes long-term survival of rat islet xenografts. Cell Transplant 17: 599–608. 18819248 10.3727/096368908786092757

[17] Muller YD, Mai G, Morel P, Serre-Beinier V, Gonelle-Gispert C, et al (2010) Anti-CD154 mAb and rapamycin induce T regulatory cell mediated tolerance in rat-to-mouse islet transplantation. PLoS ONE 5: e10352. 20436684 10.1371/journal.pone.0010352 PMC2859949

[18] Shahaf G, Moser H, Ozeri E, Mizrahi M, Abecassis A, et al.. (2011) Alpha-1-antitrypsin gene delivery reduces inflammation, increases T-regulatory cell population size and prevents islet allograft rejection. Mol Med. 10.2119/molmed.2011.00145 PMC3188864 21670848

[19] Lewis EC, Mizrahi M, Toledano M, Defelice N, Wright JL, et al (2008) alpha1-Antitrypsin monotherapy induces immune tolerance during islet allograft transplantation in mice. Proc Natl Acad Sci U S A 105: 16236–16241. 18852465 10.1073/pnas.0807627105 PMC2566995

[20] Strom TB (2005) Saving islets from allograft rejection. Proc Natl Acad Sci U S A 102: 12651–12652. 16129823 10.1073/pnas.0506079102 PMC1200297

[21] Westwell-Roper C, Dai DL, Soukhatcheva G, Potter KJ, van Rooijen N, et al (2011) IL-1 blockade attenuates islet amyloid polypeptide-induced proinflammatory cytokine release and pancreatic islet graft dysfunction. J Immunol 187: 2755–2765. 21813778 10.4049/jimmunol.1002854

[22] Schwarznau A, Hanson MS, Sperger JM, Schram BR, Danobeitia JS, et al (2009) IL-1beta receptor blockade protects islets against pro-inflammatory cytokine induced necrosis and apoptosis. J Cell Physiol 220: 341–347. 19334038 10.1002/jcp.21770 PMC2890273

[23] Lewis EC, Shapiro L, Bowers OJ, Dinarello CA (2005) Alpha1-antitrypsin monotherapy prolongs islet allograft survival in mice. Proc Natl Acad Sci U S A 102: 12153–12158. 16093309 10.1073/pnas.0505579102 PMC1189344

[24] Song S, Goudy K, Campbell-Thompson M, Wasserfall C, Scott-Jorgensen M, et al (2004) Recombinant adeno-associated virus-mediated alpha-1 antitrypsin gene therapy prevents type I diabetes in NOD mice. Gene therapy 11: 181–186. 14712302 10.1038/sj.gt.3302156

[25] Koulmanda M, Bhasin M, Hoffman L, Fan Z, Qipo A, et al (2008) Curative and beta cell regenerative effects of alpha1-antitrypsin treatment in autoimmune diabetic NOD mice. Proc Natl Acad Sci U S A 105: 16242–16247. 18852471 10.1073/pnas.0808031105 PMC2570971

[26] Hadzic R, Nita I, Tassidis H, Riesbeck K, Wingren AG, et al (2006) Alpha1-antitrypsin inhibits Moraxella catarrhalis MID protein-induced tonsillar B cell proliferation and IL-6 release. Immunol Lett 102: 141–147. 16214222 10.1016/j.imlet.2005.08.006

[27] Jeannin P, Lecoanet-Henchoz S, Delneste Y, Gauchat JF, Bonnefoy JY (1998) Alpha-1 antitrypsin up-regulates human B cell differentiation selectively into IgE- and IgG4- secreting cells. Eur J Immunol 28: 1815–1822. 9645362 10.1002/(SICI)1521-4141(199806)28:06<1815::AID-IMMU1815>3.0.CO;2-5

[28] Bergin DA, Reeves EP, Meleady P, Henry M, McElvaney OJ, et al (2010) alpha-1 Antitrypsin regulates human neutrophil chemotaxis induced by soluble immune complexes and IL-8. J Clin Invest 120: 4236–4250. 21060150 10.1172/JCI41196 PMC2993580

[29] Tilg H, Vannier E, Vachino G, Dinarello CA, Mier JW (1993) Antiinflammatory properties of hepatic acute phase proteins: preferential induction of interleukin 1 (IL-1) receptor antagonist over IL-1 beta synthesis by human peripheral blood mononuclear cells. J Exp Med 178: 1629–1636. 7693853 10.1084/jem.178.5.1629 PMC2191253

[30] Kumar R, Balhuizen A, Soni A, Amisten S, Salehi A (2011) Potential link between alpha 1 anti-trypsin and PAR-2 in the prevention of beta cell dysfunction. Mol Cell Endocrinol. 10.1016/j.mce.2011.08.040 21924322

[31] Kalis M, Kumar R, Janciauskiene S, Salehi A, Cilio CM (2010) alpha 1-antitrypsin enhances insulin secretion and prevents cytokine-mediated apoptosis in pancreatic beta-cells. Islets 2: 185–189. 21099312 10.4161/isl.2.3.11654

[32] Loganathan G, Dawra RK, Pugazhenthi S, Wiseman AC, Sanders MA, et al (2010) Culture of impure human islet fractions in the presence of alpha-1 antitrypsin prevents insulin cleavage and improves islet recovery. Transplant Proc 42: 2055–2057. 20692406 10.1016/j.transproceed.2010.05.119 PMC2924667

[33] Dhami R, Zay K, Gilks B, Porter S, Wright JL, et al (1999) Pulmonary epithelial expression of human alpha1-antitrypsin in transgenic mice results in delivery of alpha1-antitrypsin protein to the interstitium. J Mol Med 77: 377–385. 10353442 10.1007/s001090050364

[34] Subramanian S, Shahaf G, Ozeri E, Miller LM, Vandenbark AA, et al (2011) Sustained expression of circulating human alpha-1 antitrypsin reduces inflammation, increases CD4+FoxP3+ Treg cell population and prevents signs of experimental autoimmune encephalomyelitis in mice. Metab Brain Dis 2011: 25. 10.1007/s11011-011-9239-9 21437674

[35] Bellacen K, Kalay N, Ozeri E, Shahaf G, Lewis EC (2012) Revascularization of Pancreatic Islet Allografts is Enhanced by Alpha-1-Antitrypsin Under Anti-inflammatory Conditions. Cell transplantation. 10.3727/096368912X657701 23050776

[36] Mordwinkin NM, Louie SG (2007) Aralast: an alpha 1-protease inhibitor for the treatment of alpha-antitrypsin deficiency. Expert opinion on pharmacotherapy 8: 2609–2614. 17931094 10.1517/14656566.8.15.2609

[37] Arefanian H, Tredget EB, Rajotte RV, Gill RG, Korbutt GS, et al (2010) Short-term administrations of a combination of anti-LFA-1 and anti-CD154 monoclonal antibodies induce tolerance to neonatal porcine islet xenografts in mice. Diabetes 59: 958–966. 20086231 10.2337/db09-0413 PMC2844843

[38] Mandrup-Poulsen T, Zumsteg U, Reimers J, Pociot F, Morch L, et al (1993) Involvement of interleukin 1 and interleukin 1 antagonist in pancreatic beta-cell destruction in insulin-dependent diabetes mellitus. Cytokine 5: 185–191. 8218929 10.1016/1043-4666(93)90003-n

[39] Arnush M, Heitmeier MR, Scarim AL, Marino MH, Manning PT, et al (1998) IL-1 produced and released endogenously within human islets inhibits beta cell function. J Clin Invest 102: 516–526. 9691088 10.1172/JCI844 PMC508912

[40] Johansson U, Olsson A, Gabrielsson S, Nilsson B, Korsgren O (2003) Inflammatory mediators expressed in human islets of Langerhans: implications for islet transplantation. Biochem Biophys Res Commun 308: 474–479. 12914774 10.1016/s0006-291x(03)01392-5

[41] Hsu BR, Fu SH, Hsu S, Chen ST (2009) Interleukin-1 receptor antagonist enhances islet engraftment without impacting serum levels of nitrite or osteopontin. Transplant Proc 41: 1781–1785. 19545727 10.1016/j.transproceed.2008.10.099

[42] Schroppel B, Zhang N, Chen P, Chen D, Bromberg JS, et al (2005) Role of donor-derived monocyte chemoattractant protein-1 in murine islet transplantation. J Am Soc Nephrol 16: 444–451. 15601743 10.1681/ASN.2004090743

[43] Pan H, Lu HM, Hu WM, Tian BL, Liu XB, et al (2007) Anti-CD25 mAb, anti-IL2 mAb, and IL2 block tolerance induction through anti-CD154 mAb and rapamycin in xenogeneic islet transplantation. Transplant Proc 39: 3452–3454. 18089405 10.1016/j.transproceed.2007.06.091

[44] Niclauss N, Bosco D, Morel P, Giovannoni L, Berney T, et al (2011) Rapamycin impairs proliferation of transplanted islet beta cells. Transplantation 91: 714–722. 21297554 10.1097/TP.0b013e31820c10c8

[45] Chen X, Zhang Z, Su C, Gu W, Li H, et al (2010) Protective effect of heme oxygenase-1 to pancreas islet xenograft. J Surg Res 164: 336–343. 20056245 10.1016/j.jss.2009.08.016

[46] Kahraman S, Dirice E, Hapil FZ, Ertosun MG, Ozturk S, et al (2011) Tracing of islet graft survival by way of in vivo fluorescence imaging. Diabetes/metabolism research and reviews 27: 575–583. 21584921 10.1002/dmrr.1216

[47] Mordwinkin NM, Louie SG (2007) Aralast: an alpha 1-protease inhibitor for the treatment of alpha-antitrypsin deficiency. Expert Opin Pharmacother 8: 2609–2614. 17931094 10.1517/14656566.8.15.2609

[48] Karlsson-Parra A, Ridderstad A, Wallgren AC, Moller E, Ljunggren HG, et al (1996) Xenograft rejection of porcine islet-like cell clusters in normal and natural killer cell-depleted mice. Transplantation 61: 1313–1320. 8629289 10.1097/00007890-199605150-00005

[49] Deng S, Ketchum RJ, Kucher T, Weber M, Naji A, et al (1997) NK cells, macrophages, and humoral immune responses are dominant in primary nonfunction of islet grafts in the dog-to-rat xenotransplant model. Transplant Proc 29: 2062–2063. 9193527 10.1016/s0041-1345(97)00232-7

[50] Vremec D, Pooley J, Hochrein H, Wu L, Shortman K (2000) CD4 and CD8 expression by dendritic cell subtypes in mouse thymus and spleen. J Immunol 164: 2978–2986. 10706685 10.4049/jimmunol.164.6.2978

[51] Guo L, Fujino M, Kimura H, Funeshima N, Kitazawa Y, et al (2003) Simultaneous blockade of co-stimulatory signals, CD28 and ICOS, induced a stable tolerance in rat heart transplantation. Transpl Immunol 12: 41–48. 14551031 10.1016/S0966-3274(03)00016-9

